# Dominance, Politics, and Physiology: Voters' Testosterone Changes on the Night of the 2008 United States Presidential Election

**DOI:** 10.1371/journal.pone.0007543

**Published:** 2009-10-21

**Authors:** Steven J. Stanton, Jacinta C. Beehner, Ekjyot K. Saini, Cynthia M. Kuhn, Kevin S. LaBar

**Affiliations:** 1 Center for Cognitive Neuroscience, Duke University, Durham, North Carolina, United States of America; 2 Department of Anthropology, University of Michigan, Ann Arbor, Michigan, United States of America; 3 Department of Psychology, University of Michigan, Ann Arbor, Michigan, United States of America; 4 Department of Pharmacology and Cancer Biology, Duke University, Durham, North Carolina, United States of America; Indiana University, United States of America

## Abstract

**Background:**

Political elections are dominance competitions. When men win a dominance competition, their testosterone levels rise or remain stable to resist a circadian decline; and when they lose, their testosterone levels fall. However, it is unknown whether this pattern of testosterone change extends beyond interpersonal competitions to the vicarious experience of winning or losing in the context of political elections. Women's testosterone responses to dominance competition outcomes are understudied, and to date, a clear pattern of testosterone changes in response to winning and losing dominance competitions has not emerged.

**Methodology/Principal Findings:**

The present study investigated voters' testosterone responses to the outcome of the 2008 United States Presidential election. 183 participants provided multiple saliva samples before and after the winner was announced on Election Night. The results show that male Barack Obama voters (winners) had stable post-outcome testosterone levels, whereas testosterone levels dropped in male John McCain and Robert Barr voters (losers). There were no significant effects in female voters.

**Conclusions/Significance:**

The findings indicate that male voters exhibit biological responses to the realignment of a country's dominance hierarchy as if they participated in an interpersonal dominance contest.

## Introduction

Dominance contests are a critical component of determining the leadership of social hierarchies across a wide range of species [1]–[3]. In modern human societies, this dominance contest can take the form of a democratic election. Across mammalian species, testosterone is critically linked to dominance competition for hierarchical advancement in males [3]–[5]. When males win a dominance contest, their testosterone levels rise or remain stable to resist a circadian decline, and when they lose, their testosterone levels fall [3]–[5]. In men, the described pattern of testosterone change after winning or losing has been demonstrated in the context of direct, interpersonal competition (e.g., sports matches and non-physical competitions) [4], [ e.g. 6]–[8]. In addition, Bernhardt and colleagues [9] measured World Cup soccer fans' testosterone changes after the outcome of a World Cup match, and they found that vicariously-experienced competition (i.e. watching one's favorite sports teams win or lose) drives testosterone increases in winners and decreases in losers [9]. However, this single report of the vicarious-competition effect on testosterone changes is based on a small sample, tested only men, and has never been replicated [9].

What if the vicarious dominance contest is not just a sports game as tested by Bernhardt and colleagues [9], but rather it is to select the political leader of one of the most powerful countries in the world? The extent to which the described patterns of testosterone change extend to vicarious victory and defeat in broader aspects of dominance competition like political elections is unknown. Tens of millions of United States (U.S.) citizens engage in the election both directly, by voting, and vicariously, since they do not personally win or lose. This combination of direct and vicarious involvement for voters makes democratic political elections unique dominance contests. Moreover, a party-based realignment of the U.S. political leadership of profound historical significance occurs rarely. Capitalizing on this research opportunity, the present study sought to measure voters' testosterone responses to the announcement of the outcome of the 2008 United States Presidential election.

In comparison to the numerous studies of men, far fewer studies have explored women's testosterone responses to winning and losing dominance competitions. Moreover, the existing evidence is inconsistent. While a recent study has shown that winning and losing can drive differential changes in women's testosterone levels (e.g., soccer [10]), other studies have not documented this effect (e.g. rugby [11]; soccer [12]; video game [13]; computer-based cognitive game [14] (also see Archer [4] for a meta-analysis in both sexes)). Sex differences resulting from competition outcomes extend beyond differences in testosterone levels. There are also sex differences in aggression, risk-taking, and responses to threat – all behaviors which are more prevalent in men, are generally associated with testosterone increases during young adulthood, and have been shaped in male mammals through sexual selection [15]–[17]. In addition to providing more evidence in the general study of women's testosterone changes in response to competition, the present study aimed to provide the first evidence of the effects of vicarious victory and defeat on women's changes in testosterone.

We predicted that males who voted for the losing presidential candidates would have post-outcome testosterone decreases, and that the males who voted for the winning candidate would have either stable post-outcome testosterone or testosterone increases. On the basis of inconclusive but principally null findings in past research and the evolutionary perspective which suggests that testosterone plays a lesser role in female mammalian competition, we predicted that female voters would not show differential testosterone changes according to the election outcome.

## Methods

### Subjects

Data were collected from 80 participants (27 men) in Durham, North Carolina and from 103 participants (34 men) in Ann Arbor, Michigan. Eleven Durham and nine Ann Arbor participants' data were omitted from the analyses, because they did not vote or failed to complete all aspects of the experiment. The final Durham sample consisted of 69 participants (24 men) (21.07±0.46 years old). The final Ann Arbor consisted of 94 participants (33 men) (21.12±0.49 years old).

### Procedure

Participants came to the laboratory on November 3^rd^, 2008 between 10:00 am and 5:00 pm, at which point, they provided informed consent and completed a biographical questionnaire and the right-wing authoritarianism scale [18]. Participants were provided with a take-home saliva collection kit which included sampling vials, chewing gum, markers, and saliva collection instructions. On Election Night (Tuesday Nov. 4^th^), participants provided saliva sample 1 (T1) at 8pm Eastern Standard Time (EST), a time at which many election polls were closing on the east coast of the United States. Both study sites (Durham, NC and Ann Arbor, MI) are on EST. Participants provided saliva samples 2, 3, and 4 (T2, T3, T4) at 0, 20 and 40 minutes, respectively, after they had learned that Barack Obama had been declared the winner. For all samples collected at home, participants recorded the exact time of collection on the vials. On average for all participants, saliva samples 1, 2, 3, and 4 were collected at 8:08 pm, 11:35 pm, 11:57 pm, and 12:20 am, respectively (all times are EST). These times reflect participant compliance with the prescribed timing schedule of 20 minute spacing between post-outcome samples and alignment with when television networks were declaring Barack Obama the winner of the election. Participants returned their samples to the laboratory on Nov. 5^th^ between 10:00 am and 5:00 pm. On Nov. 5^th^, participants completed an endocrine health questionnaire and a retrospective affective state questionnaire. Participants also provided saliva samples at various times on Nov. 3^rd^ and Nov. 5^th^. Upon completion, participants were paid or given course credit for their participation and were debriefed. The presidential candidates on the ballots in both recruitment states used in this study were Barack Obama (Democratic party), John McCain (Republican party), and Robert Barr (Libertarian party). This study was conducted according to the principles expressed in the Declaration of Helsinki. The study was approved by the Institutional Review Board of Duke University and the University of Michigan at Ann Arbor. All participants provided written informed consent for the collection of samples and subsequent analysis.

### Self-report measures

In our retrospective affective state questionnaire, we used 9-point, Likert-scaled items to assess participants' self-reported feelings of pleasantness (unpleasant to pleasant; unhappy to happy) and dominance (dominant to submissive; controlled to controlling) at the moment when Barack Obama was declared the winner. This questionnaire also asked participants if they had consumed alcohol on the night of the election, where they viewed the election results (home, bar, campus hall, etc.), and with how many other people they viewed the election results. In our biographical data questionnaire, we also used 9-point, Likert-scaled items to assess participants' candidate support intensity (‘not at all’ to ‘as much as possible’) and participants' estimation of their candidate's likelihood of winning (‘not likely’ to ‘very likely’).

### Right-wing authoritarianism

We measured individuals' endorsement of authoritarian ideals using the right-wing authoritarianism (RWA) scale [18]–[21]. The RWA scale includes items assaying individuals' values on issues such as religion, homosexuality, abortion, marriage, feminism, moral tradition, and strong leadership. In the present samples, the 20-item RWA scale [21] showed strong internal consistency, Cronbach's α = 0.94. *Mean* = 49.67±1.58, *Max* = 114, *Min* = 20. Higher scores reflect greater conservatism.

### Salivary sampling

For each of the six saliva samples, participants used a stick of sugar-free chewing gum to facilitate collecting up to 7.5 mL of saliva in a sterile polypropylene vial and discarded the gum [22], [23]. Participants sealed the vials immediately after each collection. Participants stored their samples in refrigerators overnight. When participants returned their samples to the lab on Nov. 5^th^, the experimenter placed the samples in frozen storage. Samples were freed from mucopolysaccarides and other residuals by three freeze thaw cycles followed by centrifugation.

### Salivary testosterone

Salivary testosterone levels were assessed with solid-phase Coat-A-Count ^125^I radioimmunoassay for testosterone (Diagnostic Products Corporation, catalogue number: TKTT). This radioimmunoassay yields high correlations between salivary testosterone and free testosterone in serum in both men and women [24]–[26]. To determine salivary testosterone concentrations, we prepared water-based dilutions of all standards (with a resulting range of 5 to 400 pg/mL) and controls. 400 uL of the saliva samples, standards, and controls were pipetted into antibody-coated tubes and allowed to incubate overnight. Next, 1 ml radio-labeled testosterone tracer was added to each tube and allowed to incubate overnight. Finally, tubes were aspirated and counted for 3 minutes [23]. Assay reliability was evaluated by including control samples with known hormone concentrations in each assay (Bio-Rad Lyphochecks from Bio-Rad Laboratories, Hercules, CA). For samples of known concentration (89.7 pg/mL and 151.8 pg/mL), inter-assay CVs were 10.64% and 8.55%, respectively. Participants' six saliva samples were counted in duplicate and had a mean intra-assay CV of 14.26%. Analytical sensitivity (B_0_ -3 SD) was at 2.47 pg/mL. Mean testosterone levels for both sexes (see Table 1) are closely aligned with previous studies that employed and behaviorally validated this assay protocol [27]–[30].

**Table 1:** Sample characteristics for salivary testosterone (in pg/mL).

Salivary testosterone	Men			Women		
	Mean	SEM	CV	Mean	SEM	CV
T1	83.4	4.13	10.82%	19.5	0.86	18.21%
T2	82.6	4.78	9.60%	18.4	0.98	18.57%
T3	81.3	4.49	9.73%	16.9	0.99	17.38%
T4	76.5	4.46	9.61%	16.4	0.82	18.11%
Nov. 3^rd^	84.5	4.07	11.44%	20.3	0.95	18.53%
Nov. 5^th^	107.6	6.20	11.61%	23.2	1.22	14.42%

### Design

For the analyses, salivary testosterone on the night of the election (T1, T2, T3, T4) and self-reported mood were the dependent variables, and the 2008 Presidential candidate for whom participants voted (Obama (winner), McCain or Barr (losers)), right-wing authoritarianism, saliva collection times, and other self-report data were the independent variables. SYSTAT 12.0 statistical software was used for all analyses, with a statistical threshold of *P*<0.05. Descriptive statistics are shown as mean (± SEM).

## Results

**Figure 1:**
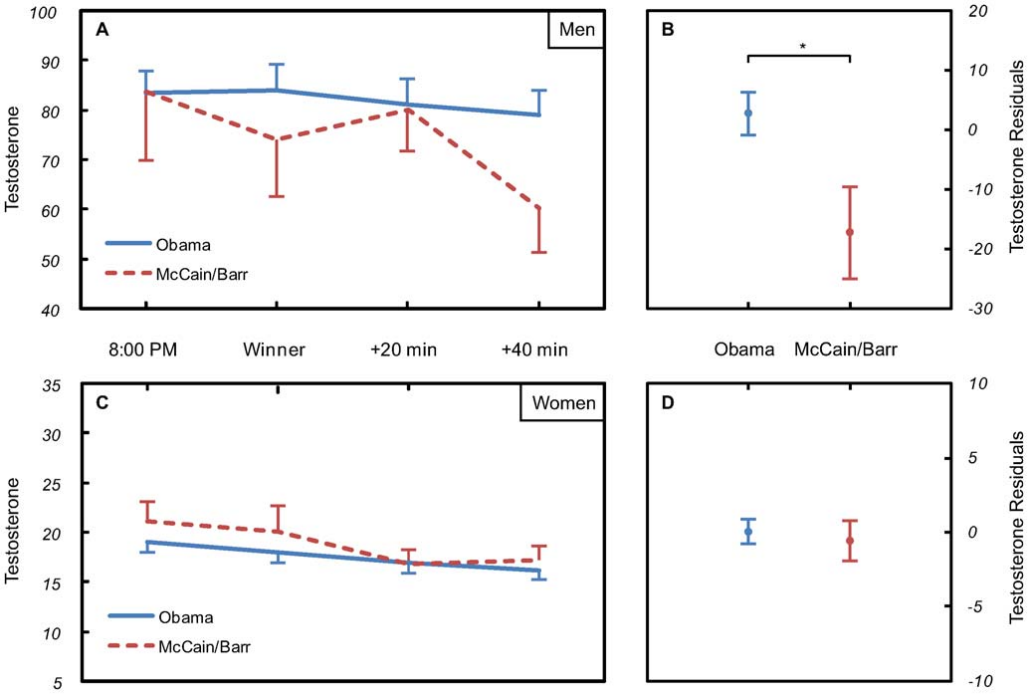
Testosterone changes on election night. Time-course of salivary testosterone (in pg/mL) in U.S. Presidential election voters on November 4^th^, 2008. In Panels A & C, times depicted correspond to T1 through T4 as described in the paper. Testosterone residual change scores from T1 to T4 in men (Panel B) and women (Panel D) who voted for the winner (Obama) or the losers (McCain or Barr).

To examine the impact of candidate choice on testosterone levels after the election outcome announcement, a repeated-measures ANCOVA was run with post-outcome testosterone at T2, T3, and T4 as a within-subjects factor and testosterone at T1 as a baseline covariate. A significant Time x Outcome (Win/Loss) interaction was observed in men (*F*(2, 100) = 3.40, *p* = 0.04), but not women (*F*(2, 188) = 0.39, *p* = 0.68) (Fig. 1). To quantify the effect of the outcome on relative changes in testosterone from before to after the election, residualized testosterone change scores were calculated from T1 to T4, where effects were predicted to be maximal according to time-course changes in salivary testosterone [31]. Residual testosterone change scores measure testosterone change between two time-points (T1 & T4) while controlling for variance in testosterone at baseline (T1). Using ANOVA, candidate choice predicted differences in men's testosterone residuals (*F*(1, 51) = 4.72, *p* = 0.03), with supporters of John McCain or Bob Barr having significantly larger testosterone decreases from T1 to T4 than supporters of Barack Obama (Fig. 1). Moreover, the candidate choice effect on men's testosterone change remained even when participants' conservatism, as measured by the RWA scale [18], was partialled out of the analysis (*F*(1, 49), = 5.39, *p* = 0.03). Further still, the candidate choice effect was maintained when adding an additional covariate which accounted for voters' intensity of support for their candidate (*F*(1, 48), = 5.37, *p* = 0.03). Using ANOVA, voter group failed to predict differences in women's testosterone residuals (*F*(1, 97) = 0.12, *p* = 0.74) (Fig. 1). When including the RWA scale and voters' support intensity as covariates, voter group still failed to predict differences in women's testosterone residuals (*F*(1,91) = 0.71, *p* = 0.71).

We also repeated the analyses excluding the participants who voted for Robert Barr, who arguably did not have a chance of winning. The results were essentially unchanged. A significant Time x Win/Loss interaction was still observed in men (*F*(2, 98) = 3.94, *p* = 0.02), but not women (*F*(2, 186) = 0.22, *p* = 0.80). Voter group still predicted differences in men's testosterone residuals (*F*(1, 50) = 4.94, *p* = 0.03), including when differences in participants' conservatism was partialled out (*F*(1, 48), = 5.94, *p* = 0.02) and when voter support intensity was also partialled out (*F*(1, 47), = 5.81, *p* = 0.02). Voter group still failed to predict differences in women's testosterone residuals (*F*(1, 96) = 0.00, *p* = 0.97).

We wanted to rule out other factors that might have contributed to men's changes in testosterone levels. To do so, we examined the effects of male participants' social surroundings and alcohol consumption on the evening of the election on their testosterone responses. We examined the effect of alcohol consumption, because alcohol consumption can lead to decrements in testosterone in men [32]. We performed a repeated-measures ANCOVA with post-outcome testosterone at T2, T3, and T4 as a within-subjects factor and testosterone at T1 as a baseline covariate, and also added covariates that accounted for where the participants viewed the election (home, bar, campus hall, etc.), with how many people they viewed the election, and whether or not they consumed alcohol on the night of the election. None of these factors absorbed a significant portion of the variance (all *F*s<1.0), and the Time x Outcome (Win/Loss) interaction was still significant and of the same magnitude (*F*(2, 94) = 3.27, *p* = 0.04). We also included the same three covariates in an ANOVA testing the effect of candidate choice on testosterone residuals. Again, these factors failed to absorb a significant portion of the variance (all *F*s<1.0), and candidate choice significantly predicted the difference in testosterone residuals (*F*(1, 48) = 4.45, *p* = 0.04), with supporters of John McCain or Bob Barr having significantly larger testosterone decreases from T1 to T4 than supporters of Barack Obama. In female voters, we also confirmed that these factors (alcohol consumption, social setting, number of co-viewers) failed to account for a significant portion of the variance in the Time x Outcome repeated-measures ANCOVA and the test of candidate choice on testosterone residuals.

Lastly, for men, we also wanted to rule out the influence of the timing of post-outcome saliva collection on testosterone change. To do so, we performed a repeated-measures ANCOVA with post-outcome testosterone at T2, T3, and T4 as a within-subjects factor and testosterone at T1 as a baseline covariate and added the time of day at T2, T3, and T4 as covariates. The Time x Outcome (Win/Loss) interaction was still significant (*F*(2, 84) = 3.21, *p* = 0.05). Moreover, candidate choice still predicted differences in men's testosterone residuals (*F*(1, 43) = 4.39, *p* = 0.04). These results suggest that variation in saliva collection times did not alter the effect of candidate choice on testosterone change. We repeated these analyses with time covariates for women, and we still failed to find a Time x Outcome (Win/Loss) interaction (*F*(2, 158) = 0.83, *p* = 0.83) or a candidate choice effect on women's testosterone residuals (*F*(1, 83) = 0.08, *p* = 0.78).

In order to address potential explanations of the sex differences in testosterone responses, we tested sex differences in participants' candidate support intensity, their levels of right-wing authoritarianism, their estimates of “their” candidate's likelihood of winning the election, their consumption of alcohol, and their social surroundings on the night of the election. There were no differences between the sexes in their candidate support intensity (*t*(161) = 0.30, *p* = 0.77), their estimates of “their” candidate's likelihood to win the election (*t*(161) = 1.12, *p* = 0.27), their degree of right-wing authoritarianism (*t*(155) = −0.06, *p* = 0.96), their consumption of alcohol (*t*(161) = −0.853, *p* = 0.40), the type of social setting where they watched the election (*F*(1, 159) = 0.11), *p* = 0.74), or the number of people with whom they watched the election (*F*(1, 159) = 0.05), *p* = 0.82).

In retrospective reports of their affective state upon the announcement of Obama as the president-elect, McCain and Barr voters felt significantly more unhappy (*t*(159) = 22.98, *p*<0.001), submissive (*t*(160) = −11.30, *p*<0.001), unpleasant (*t*(160) = −20.10, *p*<0.001), and controlled (*t*(158) = 6.42, *p*<0.001) than Obama voters.

### Discussion

While past studies have shown that men's testosterone levels differentially change in response to winning or losing an interpersonal dominance contest, the present study provides novel evidence showing that vicarious victory and defeat via democratic elections has similar physiological consequences for male voters as do interpersonal dominance contests [5]. Confirming our first hypothesis, we found that men who voted for Barack Obama (winner) had stable post-outcome levels of testosterone, and men who voted for John McCain or Bob Barr (losers) had decrements in their testosterone levels. Moreover, the pattern of testosterone change remained significant even when variance in a multitude of factors was controlled for including voters' political values, support intensity for their candidates, timing of saliva collection, levels of conservatism, consumption of alcohol on the night of the election, and social surroundings on the night of the election. The robust nature of the statistical effect, even when accounting for several potential explanatory factors, strongly implicates a win/loss effect on testosterone change.

Voters experienced the outcome of winning or losing vicariously through their candidate. While voting involves direct participation in the electoral process, voters don't personally win or lose the election. In this regard, the present results are similar to the study by Bernhardt and colleagues [9] which showed that male sports fans' testosterone levels changed according to whether their team won or lost [9]. Thus, the present data offer the first empirical support of the findings of Bernhardt and colleagues [9]. The present data also extend hormonal analysis of vicarious victory and defeat to the sociopolitical domain, which had not been tested heretofore.

In past studies of competition, males' testosterone levels have risen in response to winning [4]. However, in this study, winning males' testosterone stayed constant from four hours before to after the election outcome, as opposed to rising. We argue that this is evidence of resistance to the circadian decline in men's testosterone levels, which would typically be observable over the four hour period spanning election polls closing to the collection of the last post-outcome saliva sample [22], [25]. Thus, such resistance to circadian decline over such a long period is conceptually similar to a rise in testosterone levels. Most previous non-physical competition studies have used competitions with significantly shorter durations [4], [27], [31], [33], during which testosterone levels would be much less susceptible to circadian changes. In contrast, the observed drop in the salivary testosterone levels of McCain and Barr voters was of greater magnitude than would be expected as a function of circadian decline [22], [25]. In an effort to specifically control for the effects of normal circadian decline in testosterone levels over several hour spans, future studies of this nature could also collect saliva samples from the same subjects on a control evening over the same span of time.

In confirmation of our second hypothesis, we found that female voters' testosterone levels did not change as a function of the election outcome. As measured by self-reported intensity of support for their candidate, female voters wanted their presidential candidate to win as much as male voters did, and they thought that their candidates were equally likely to win as did male voters. In addition, female voters were not different from male voters in their levels of conservatism. This evidence supports the conclusion that the observed sex difference in testosterone responses was not driven by variance in political zeal or values. In addition, the present data suggest that there is a sex difference in testosterone responses to vicariously-experienced dominance contests, which had previously been reported only in men [9]. The few studies on women's testosterone responses to winning and losing a competition have failed to present a consistent set of results [4], [10]–[14], and the present study adds evidence in support of the existing null findings. It is more difficult to measure salivary testosterone accurately in women than in men, and this could have contributed to the null finding in women [34]. Moreover, the biological mechanism that mediates males' rapid testosterone changes (via the testes) in response to winning and losing does not have a well-researched parallel mechanism in females (via the ovaries and adrenal glands) [31], [35], [36]. In combination, these factors may explain the null finding in women from both methodological and biological perspectives.

Physiological changes in voters were also accompanied by changes in affective state. Those who voted for a losing candidate felt significantly more controlled, submissive, unhappy, and unpleasant at the moment of the outcome than did those who voted for the winning candidate, which corroborates past research [31]. However, it is unclear the extent to which testosterone is directly implicated in these subjective affective states. In humans and other mammals, males' testosterone increases after winning promote willingness to compete in another dominance contest, while testosterone decreases promote withdrawing from further competition [3], [33], [37]. Since losing voters reported greater submissiveness, we speculate that losing males, who also experienced testosterone decrements, might have been less motivated to engage in dominance behavior after the election. Moreover, since the dominance hierarchy shift following a presidential election is stable for 4 years, the stress of having one's political party lose control of executive policy decisions could plausibly lead to continued testosterone suppression in males [2].

The present study focused on one aspect of cultural dominance – the re-establishment of a social hierarchy by a democratic election of a national leader. It is unknown whether shifts in international political dominance (e.g., winning or losing wars), business power (e.g., the outcome of labor union negotiations), or economic strength (e.g., events of economic boom and bust) also drive changes in citizens' physiology. As in the election, these macro-sociological events also differ from micro-sociological face-to-face dominance competitions, because the outcomes are vicariously experienced by members of the participating groups. Future research could also directly test the candidates' endocrine responses to political elections outcomes, which would be more directly analogous to social dominance contests in non-human primates.

To conclude, the present results suggest that male, but not female, voters respond with testosterone changes to the outcome of presidential elections as if they had personally fought to ascend a social dominance hierarchy. In his victory speech, Barack Obama said, “…I will never forget who this victory belongs to, it belongs to [Obama voters],” and male voters' testosterone levels reflected his sentiments regarding winning the dominance contest that is the U.S. Presidential election.
